# Acceptability, Usability and Weight Loss Outcomes in a Randomized Cross-Over Study of Commercially Available Portion Size Tools in an Overweight South Asian Community

**DOI:** 10.3390/ijerph19137714

**Published:** 2022-06-23

**Authors:** Basma Ellahi, Amanda Aitken, Derya Dikmen, Bilge Seyhan-Erdoğan, Munibah Makda, Rifat Razaq

**Affiliations:** 1 Faculty of Health and Social Care, University of Chester, Parkgate Road, Chester CH14BJ, UK; a.aitken@chester.ac.uk (A.A.); munibah-m@hotmail.com (M.M.); rifatrazaq@gmail.com (R.R.); 2 Department of Nutrition and Dietetics, Hacettepe University, Ankara 06230, Turkey; ddikmen@hacettepe.edu.tr (D.D.); bilgeseyhan6@gmail.com (B.S.-E.)

**Keywords:** portion size, portion control tools, migrant groups, weight loss, dietary change, co-creation

## Abstract

South Asian women living in the UK are particularly at high risk of obesity-related complications, such as type 2 diabetes and cardiovascular disease. Exposure to large portion sizes is a risk factor for obesity. Specifically designed tableware helps individuals to manage weight by controlling food portion sizes. Thirty-one (*n* = 31) overweight or obese South Asian adult women participated in a randomised cross-over trial aimed to assess the efficacy, acceptance, and weight change of two guided/calibrated commercially available portion control tools (Utensil set and Crockery Set) used in free-living conditions. Data on acceptance, perceived changes in portion size, frequency, and meal type was collected using paper questionnaires and 3-day diet diaries. Scores describing acceptance, ease of use, and perceived effectiveness were derived from five-point Likert scales from which binary indicators (high/low) were analysed for significance using multivariate variance analysis for repeated measurements. A reduction in BMI was observed at each point of measurement (*p* = 0.007). For overall tool use, the crockery set scored higher in all areas of acceptance, ease of use, and perceived efficacy for all comparisons. Self-selected portion sizes increased for salads and decreased for cooking oil and breakfast cereals with both tools. Further research to scale up and evaluate similar weight management interventions for this group is warranted.

## 1. Introduction

South Asians are the largest migrant ethnic minority in the UK, representing 7.5% of the total population [1]. Recent NHS England data indicates the proportion of overweight and obese South Asians (SA) in the UK increased to 59.7% in 2019/20 [2]. South Asian women living in the UK are particularly at high risk of obesity-related complications, such as type 2 diabetes (T2D) and cardiovascular disease [3,4]. Furthermore, in a study comparing SAs to their host Europeans, authors reported that SAs’ risk of T2D is three to five times higher [5] and the development of T2D occurs typically 5–10 years earlier and at lower BMIs [6]. People with T2D tend to be overweight or obese and therefore the current recommended treatment strategy is weight loss (through diet changes) and exercise [7,8], although this advice is based largely upon evidence from studies in non-South Asian populations [9]. Furthermore, data from the COVID-19 pandemic in the UK showed an ethnic risk of COVID-19 mortality that was dependant on BMI. The estimated risk of COVID-19 mortality at a BMI of 40 kg/m^2^ in white ethnicities was equivalent to the risk observed at a BMI of 27.0 kg/m^2^, in the South Asian minority group [10].

Reported barriers to weight loss in the South Asian community include the influence of religion, culture, and family expectations on home cooking, perceptions that weight gain is inevitable (owing to ageing, childbirth, or divine predestination), and the prioritisation of family concerns over individual lifestyle change [11]. Additionally, the dietary habits of South Asian men and women (aged 26–67) are also strongly affected by socio-cultural factors such as language and literacy barriers, health knowledge, and the level of acculturation [12].

Eating smaller portions is recommended as a weight control strategy. However, adequate portion size estimation is necessary to achieve good portion control during weight loss and maintenance [13]. Findings from two trials in the UK suggested that a portion control intervention using a portion control plate and dietary counselling may be effective for enhancing weight loss among obese subjects [14,15]. However, the portion size plates are designed for generic foods and may not be as useful for an Asian diet with largely amorphous foods. A major challenge is the accurate estimation of portion sizes for traditionally consumed foods when there are few specific validated dietary assessment tools for this population [16]. Initial findings from a systematic review in our group have revealed that the use of portion size tools in ethnic populations has been considerably underexplored [16]. Furthermore, successful weight loss interventions for this South Asian community group are not widely reported and dieters experience many challenges [17]. Calibrated tableware may be potentially effective instruments for inclusion as part of weight loss interventions [18]. Thus, a focus on portion control may be an effective dietary approach, which is easy to implement. Given the known barriers to weight loss in this community, we aimed to investigate the efficacy and acceptance of two commercially available calibrated portion control elements (or tools) in a sample of overweight or obese South Asian women, when used in free-living conditions as an aid to portion control and weight loss (for the tools and overall weight loss), with minimal health professional contact. We hypothesised there will be no differences between the two sets of portion control tools; the study group would demonstrate more control of their eating behaviours, and that weight would stay the same or there would be modest weight loss over the study period.

## 2. Materials and Methods

The research approach was co-created with a sample of people from the South Asian community not included in the final study sample. This comprised of researchers and community contacts (lay members) recruited from the same South Asian community. This enabled a focus on the aspects important to this community, established the research protocol and the recruitment strategy.

### 2.1. Recruitment and Eligibility

South Asian women aged 18 and over were recruited in Greater Manchester (Areas 1 and 2) Lancashire (Area 3) and Leeds (Area 4) all in the North of England during 2017 and 2019 avoiding Ramadan periods during the summer. The recruitment strategy used posters, e-posters, and word of mouth through existing community contacts and networks. Interested candidates were invited to recruitment meetings where further information was provided, including a participant information sheet and a questionnaire (to gather screening and baseline data). Women with a BMI of >23 kg/m^2^ of self-reported South Asian ethnicity and who met the inclusion criteria and wanted to lose weight or maintain weight were enrolled in the study. The lower cut-off point for the BMI rather than 25 kg/m^2^ is used as this value is an indicator of the risk of NCDs in this group [19,20]. This use is further supported by a recent study where the authors conclude appropriate clinical surveillance to optimise the prevention, early diagnosis, and timely management of type 2 diabetes is required using these cut-off points for risk [10]. Earlier phase 1 research participants, reported elsewhere [21] were excluded, as were males, and those already on a commercial weight loss diet, previously or presently using tools to measure portion size. Those suffering from active cancer, pregnant or lactating, or been diagnosed with an eating disorder, mental illness, or psychiatric disorder within the last 12 months that required active treatment were also excluded (*n* = 17). In this study, these were mainly women who were taking prescribed antidepressants.

### 2.2. Study Design and Portion Control Tools

**Figure 1:**
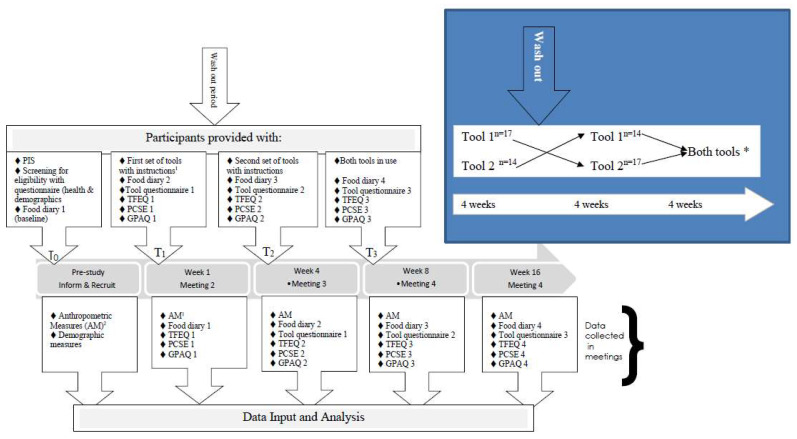
Crossover study protocol and design. 1 Portion size tools were allocated randomly. 2 Anthropometric measures were taken to confirm BMI > 23 kg/m^2^ for eligibility and at week 1 for a baseline measure. Key = Participant Information Sheet (PIS), Anthropometric Measures (AM), Three Factor Eating Questionnaire (TFEQ), Portion Control Self-Efficacy Questionnaire (PCSE), Global Physical Activity Questionnaire (GPAQ) Tool 1 = Crockery Set, Tool 2 = Utensils and BOTH tools * the participants choose from the two sets their preference T_0_, T_1_, T_2_, T_3_ are intervention points.

The study utilised a crossover design [22], with minimal health professional contact, outlined in Figure 1. All research assistants involved with data collection were trained on the methods to ensure consistency between measures. Specific training on translations of all data collection tools was provided to assure quality. Enrolled participants provided written consent. Participants were provided with a three-day self-reported food diary for completion at home in the week prior to attending the first data collection meeting of the study at a community centre, usually one to two weeks later, at which the completed diary (dairy 1 T_0_) was collected, and anthropometric measures were re-taken (Figure 1). Diary data included one weekend day and two other typical days of the week. Height without shoes was measured using stadiometer (SECA 875) to the nearest 0.1 cm, and weight (without shoes) was measured using a digital scale (SECA 217) to the nearest 0.1 kg. Waist circumference measures were taken through light clothing using a non-extensible measuring tape to the nearest 0.1 cm using CDC (2007) criteria [23]. During this meeting, the Three Factor Eating Questionnaire (TFEQ) [24], the Portion Control Self-efficacy Scale questionnaire (PCSE) [25], and a global physical activity questionnaire (GPAQ) [26] were completed by participants with assistance from trained translators as necessary.

**Figure 2:**
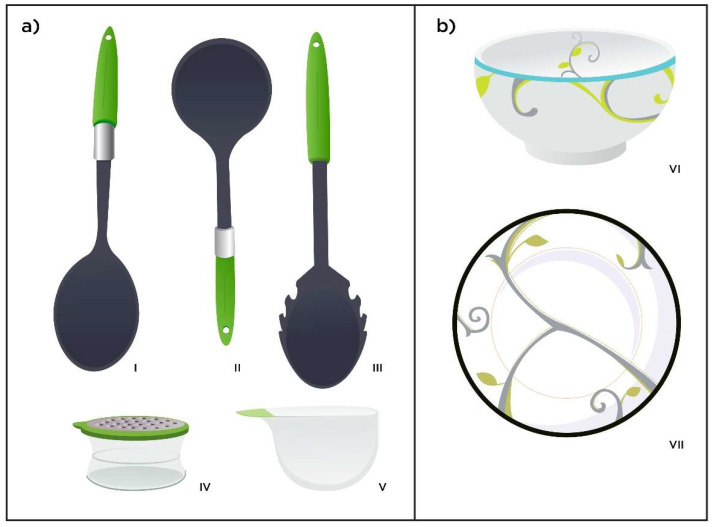
Photo (**a**) utensil set, (**b**) crockery set. The plastic serving utensils set (**a**) comprised of: a serving spoon (**i**) with capacity for 1 portion of starch (1/2 cup); a ladle (**ii**) for cream-based sauce or gravy; pasta server (**iii**), 2 spoons = 1 serving; a cheese grater (**iv**) the first line for one serving or to the top for two and a cereal scoop (**v**) one serving (1 cup/25 g) of ready to eat cereal. The crockery set consists of a bowl (**vi**) and plate (**vii**).

Two sets of guided calibrated portion control tools Utensil set (US) and Crockery Set (CS) (Figure 2) were used in the study selected through focus groups (phase 1) [21]. Both were accompanied by a set of simple instructions based on the manufacturer’s information, including details on each component of the tool set (i.e., crockery or utensil set) and information on how each tool corresponds to reference portion sizes. For example, with the utensils set the ladle is recommended for soup, sauce, or gravy items: For cream-based sauce or gravy fill to the bottom gravy line, for a non-cream sauce fill to the middle sauce line for soup fill to the top mark (1 cup/1 serving). Very simple instructions were provided which encouraged a balanced plate along the lines of the Eatwell guide [27]. (Instructions are available in Supplementary Information (File S1a and S1b; File S2a and S2b)). The women were encouraged to decrease consumption of energy-dense foods (including high fat and sugar foods) and high salt foods and increase consumption of fruit and vegetables as this was flagged up from their initial diary data.

All measures are based on United States Department of Agriculture standards [28]. The ladle and the cereal scoop portion sizes are based on FDA food labelling requirements [29]. Supplementary Information File S3 provides a comparison of the USA portion sizes with the UK ones).

The crockery set (b) a crockery bowl (vi) with disguised marks for 1/2, 1, 1.5 cup and a 9-inch (23 cm) plate (vii) decorated with leaves depicting three sectors discretely (non-starchy vegetables; protein and starch, which includes high fibre carbohydrate foods) [30].

Participants were randomly allocated (using sealed envelopes a and b on a 1:1 allocation) to receive the first portion control tool either the utensils (a) or the crockery set (b) with the instructions. Seventeen participants used the utensils set (US) first and fourteen the crockery set (CS) first and then crossed over (as per Figure 1). Participants also received a study questionnaire for the first tool (comprised of closed and open-ended questions) and a three-day food diary 2 (T_1_ data collection) (Figure 1). Participants were introduced to the tool and asked to use the tool for 4 weeks and complete the study questionnaire, whilst using the tool, in week 3 of the 4-week period. The completed first set of data collection tools was collected at meeting 2 where TFEQ, PCSE, and GPAQ questionnaires were completed, and anthropometric measures were taken. A washout period of two weeks was undertaken before the above was repeated for the use of the second set of tools (T_2_). For the third meeting data was collected as per the previous meetings, but participants were instructed to use preferred items from both sets of tools for an eight-week period with a study questionnaire assessing the use of the tools of their choice provided. Thus, each participant was asked to use each of the two set of tools separately for 4 weeks and then for the last 8 weeks use preferred items from both sets with the order of use assigned randomly and with minimal health professional contact (T_3_). The total testing period was 16 weeks, excluding the washout period. A WhatsApp (social media) group was used to increase motivation where sharing pictures of meals was undertaken voluntarily, and regular telephone reminders were undertaken by research assistants to aid data collection. A verbal debrief was undertaken at the end of the study with the participants. Tool questionnaires are available in Supplementary Information (File S1a and S1b).

### 2.3. Sample Size

Based on previous research [31], one can estimate that 30 participants would be sufficient to collect information on the tools’ effectiveness and acceptance at a descriptive level. To account for an expected dropout rate of 50% from start to end plus a further 20% dropping between T_1_ and T_2_ due to carry-over effects a total of 75 participants needed to be recruited.

### 2.4. Data Collection Tools

#### 2.4.1. Tool Usage

A paper copy of a semi-structured multiple-choice study questionnaire (File S1a and S1b) completed by participants was used to assess tool usage. This included frequency of tool use, type of meal, and self-reported change in portion size relating to food groups. Quantitative outcome data on the acceptability of the portion size tool set, ease of use, and perceived effectiveness of the tool in controlling portion size was collected, mainly using Likert scale questions. A 100 mm visual analogue scale (VAS) assessed the likelihood of anticipated adherence for the tool with a scale from ‘very unlikely’ to ‘extremely likely’, from which means scores were calculated (S1). The study tool questionnaire used was developed from earlier work by [32] and was deemed to have good content and face validity.

#### 2.4.2. Physical Activity

The shortened validated global physical activity questionnaire (GPAQ) [26] was used to categorise the participants’ self-reported physical activity levels into inactive, moderately inactive, moderately active, and active. The results were coded on a scale from 1–4 with 1 = active. The data was used to establish whether activity levels had increased, decreased, fluctuated, or remained the same throughout the study.

#### 2.4.3. Eating Behaviours and Appetite

The Three-Factor Eating Questionnaire (TFEQ) is a validated self-assessment scale used widely in studies of eating behaviour in overweight and normal-weight individuals. It was designed to assess three cognitive and behavioural domains (or ‘factors’) of eating: cognitive restraint (CR), disinhibition, and hunger [24]. The definitions for these aspects are as follows:

Dietary restraint (DR) is a tendency to consciously restrict or control food intake (possible score 21). Thus, the higher the score the better the self-control.

Dietary disinhibition (DD) is a tendency to overeat in the presence of palatable foods or other disinhibiting stimuli, such as emotional stress (possible score 21). Thus, the lower the score the better the control.

Hunger (H) is characterized by its internal (regular feeling of hunger interpreted and regulated internally) and external traits (feeling of hunger triggered by external cues (social occasions, for instance) (possible score 14). Thus, the higher the score the hungrier the person feels [24].

The Portion Control Self-Efficacy (PCSE) tool was used to assess a reliable and valid measure of self-efficacy towards portion control on eating behaviours and subsequent weight-loss and health outcomes [25].

#### 2.4.4. Diet Diaries

The participants were asked to complete 3-day food diaries a total of four times; at baseline, at week three, week seven, and week fifteen, the last week of the tool usage (excluding washout period). Diaries were distributed and collected at each meeting (Figure 1).

#### 2.4.5. Ethics

This study was conducted according to the guidelines laid down in the Declaration of Helsinki. The research protocol and procedures were approved by the Faculty of Health and Social Care Research Ethics Sub-Committee. Written informed consent was obtained from all participants and those that completed the study had the option to keep the tools they used.

#### 2.4.6. Data Input and Statistical Analysis

Statistical analyses were performed using the Statistics Program for Social Sciences software (SPSS Statistics for Windows version 24, SPSS Inc., Chicago, IL, USA). Levene’s test of normality, and the Shapiro–Wilk test were used to check the normality of the variables. Nominal data were examined using a chi-squared test and continuous (ordinal) data were presented as means, standard deviation, and range.

The difference in anthropometric measurements (BMI, weight, and waist circumference) according to tool usage were determined with an analysis of variance in repeated measures. In order to examine which tool was efficient for resultant weight, BMI, and waist circumference change, an independent *t*-test was used. The one-way ANOVA test was used for comparison of more than two group means. Differences were considered significant at *p* < 0.05. Analysis of variance in repeated measures test was used to check whether physical activity changed over time.

The mean TFEQ scores were calculated using the author’s instructions [24]. Tool acceptance was the mean score of questions 4a, 4b, 4c, and reverse of 4d (liking, fitting the kitchen, fitting with home life, and not feeling embarrassed using it), ease of use was calculated 4e, 4f, and 4g (easy use, resistant to wear and tear, and having clear instructions, ease of use compared to other tools) and perceived efficacy was the mean score of questions 4h, 4i, 4j, and 4k. A score of 0 to 2.5 was considered a ‘none–too low’ result, a score between 2.6 to 3.4 as ‘neutral’, and between ‘3.5 to 5 to’ considered as moderate to high. The mean score for acceptance, ease of use, and effectiveness and mean physical activity levels were tested for significance using multivariate variance analysis for repeated measurements.

Qualitative comments and comments received during the de-briefing were also gathered and the consensus was reported.

The diet diaries were examined qualitatively assessing common foods eaten and changes over the intervention. Completed diet diaries were inputted into Microdiet (Downlee Systems Ltd. Version 4, High Peak Derbeyshire, UK) and analysed for mean energy intake and macronutrients for each participant. For calculating BMR, the Schofield equation was used [33]. Under- or over-reporting of dietary intake was calculated as the ratio of energy intake (EI) to Basal Metabolic Rate (BMR) according to the method of [34]. This ratio was calculated for each individual and the cut-off values for EI:BMR used were (i) <1.14 and between 1.14 and 1.34 were classified as under-reporters, (ii) if the ratio was between 1.35 and 2.39 were classified as normal reporters and (iii) if it was >2.4, they were classified as over-reporters. Number of calories under-reported was also calculated.

The following outcome measures were assessed during data analysis:

- Change in weight (kg), BMI, and waist circumference (cm) from baseline to 4, 8, and 16 weeks, respectively. Changes due to the tool used. No weight change, no increase in weight, or decrease in weight (weight loss) were considered as measures of success.
- Efficacy as a change in energy intake from baseline (measured by diet diary analysis).
- Change in diet composition at 4, 8, and 16 weeks from the baseline (from diet diaries) and from Question 6 from Questionnaires 1, 2, and 3.
- Change in physical activity over the study period (although no change is desirable).
- Change in the portion control and self-efficacy scale (PCSE) score (PCSE at 4, 8, and 16 weeks T_1_, _2_, and _3_, respectively, from baseline T_0_ according to Fast et al. [25].
- Changes in dietary restraint, disinhibition, and hunger in relation to tool use across the study (TFEQ at three time points) at 4, 8, and 16 weeks T_1_, _2_, and _3_ from baseline T_0_.
- Acceptance levels of each portion tool (Questionnaires T_1_ and T_2_).
- Ease of use of each portion tool (Questionnaires T_1_ and _2_).
- Perceived efficacy of each portion tool (Questionnaires T_1_ and T_2_).
- Acceptance, ease of use and perceived efficacy and likeability of both tools and their comparison (Questionnaire T_3_).

## 3. Results

### 3.1. Baseline Characteristics

**Figure 3:**
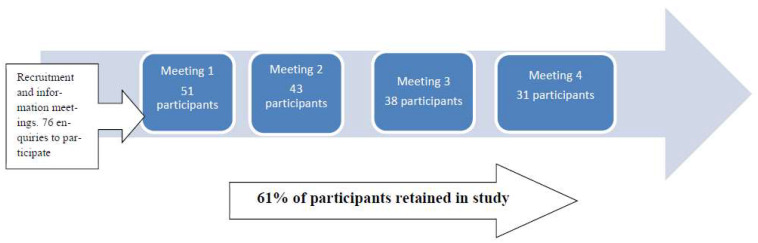
Recruitment and retention of participants.

Thirty-one women completed the trial by attending all 4 meetings, with an overall retention rate of 61% (Figure 3). Seventeen women were excluded at the screening stage. Seven participants were from South Manchester (Area 1), six participants from North Manchester (Area 2), nine from Blackburn (Area 3), and nine from Leeds (Area 4). Most of the women (64%) were between the age of 30 and 50 years old. Area 1 and Area 2 had the highest number of older participants, with 3 and 2 participants, respectively, over 50 years old. Area 3 had the highest number of younger participants with 4 participants under the age of 30, (data not shown). The majority of participants were married (89.3%), had completed high school (58%), and were parents (80.6%), with a mean number of 2.7 children (Table 1). The majority of those in Area 1 and Area 3 classified themselves as Asian British, whereas others classified themselves as simply Asian. All women originated from South Asian countries and were Muslim. All participants had BMIs over 23 which according to NICE guidelines places them in a risk category for comorbidities such as type 2 diabetes [35] (reported later).

### 3.2. Meal Pattern and Consumption

Eighty-four percent of the women reported consuming three meals a day and the remainder 2–3 meals a day, with 74% of all women consuming breakfast daily, Fifty-eight percent of participants consumed homemade meals between 4 and 6 times a week, and 77% consumed food from takeaways between 1 and 3 times a week. (Table 2). The majority of the participants were the main shopper and main meal preparers for their homes; at sixty-eight percent and eighty-one percent, respectively (data not shown).

### 3.3. Acceptance, Ease of Use, Perceived Efficacy and Likelihood of Continued Use

The majority of participants reported that the utensils and the crockery set aided portion self-served, self-control, particularly of starchy foods, and aided learning about portions.

For overall tool use, the crockery set scored higher in all areas of acceptance, ease of use, and perceived efficacy (data from questionnaires 1 and 2), (Table 3).

Self-reported modifications for preparation, serving or consumption of usual portion sizes of the main food groups (data from Q1, 2, and 3) are reported in Table 4. Overall vegetable and salad consumption had increased during the study whilst a decreased overall consumption of rice, potatoes, chips/roast potatoes, pasta, and cheese were noted (Table 4). This reflects a focus on reducing carbohydrates and high-fat foods. For the utensils ‘increased’ consumption was observed for vegetables and salad. For the crockery set, “increased” consumption was observed for salads. Individual differences in the use of the two-portion control tools were observed as noted in Table 4. These differences relate to the usability and versatility of the tools for food groups and foods commonly consumed by Asians, discussed later.

Data from the qualitative comments made by participants in the questionnaire (File S1a and S1b) and oral feedback in a debrief at the end of the study indicates the utensils were not deemed as acceptable, useable, or effective at the table compared to the crockery set. Utensils were reported to be ‘clumsy to bring to the table’ as the serving spoons that are typically used at the table would be quite different from the utensils provided, the latter being more visually attractive and more suitable for the typical serving size of the type of food being served. Some of the utensils were deemed too large for the dish being served and therefore not useful in a dining setting. However, for single-person usage in the kitchen, they were deemed acceptable. This explains why they were less used across the sample as most participants were living with family. Utensils were deemed to be more useful if you are serving food from the kitchen and delivering the plates to the table for consumption.

The crockery set on the other hand was visually more acceptable and participants reported they found it easier to judge portion size when using them. It was deemed as “discrete” since the quality of the product fitted in with household crockery better. From the crockery set, the plate was reportedly used to self-assess portions and aided in reducing carbohydrate foods and increasing vegetables and salad.

### 3.4. Weight, BMI, and Waist Circumference

Changes in weight, BMI, and waist circumference were observed over the duration of the study. Nineteen participants lost weight, five showed no overall weight change and only seven gained weight. Area 2 had the largest weight, BMI, and waist circumference at baseline. Area 1 had the largest variation of weight (range) and BMI. Area 2 had the largest variation (range) in waist circumference at the start of the study (data not shown).

Overall, the mean weight, BMI, and waist circumference decreased from baseline (week 1) to the end of the study (week 16), with mean changes of −0.97 kg (SD 1.74), −0.35 kg/m^2^, and −1.52 cm, respectively, (Table 5). Statistically significant differences were observed for weight, between 1st measurement (week 1/baseline) and 4th measurement (week 16/last) (*p* = 0.026) and 2nd (week 4) measurement and 4th (week 16/last) (*p* = 0.009). The change in BMI was statistically significant at all four data collection points i.e., a reduction in BMI was observed at each point of measurement (*p* = 0.007). For waist circumference, this was a 1.52 cm reduction overall (*p* = 0.049), but there was no statistical difference over time (Table 5).

There was no change in these parameters due to the type of tool used and order. A mean weight loss using the crockery set of −0.22 kg was observed and for the utensils, this was −0.26 kg (calculated over weeks 1–4 and 5–8. No statistically significant difference was observed between BMI and the type of tool used first or second i.e., crockery or utensils and vice versa (results from an independent *t*-test *p* = 0.094).

### 3.5. Education Level and Weight Change

There was no statistically significant difference between change in weight and education level (ANOVA test *p* = 0.114, data not shown).

### 3.6. Eating out and BMI Change

The majority of the women (61%) reported that they ate out (non-home cooked food) between 1 and 3 days a month, 29% of women ate out less than once a month and 9.7% ate out between 4 and 6 days a month. The women that ate out less than once a month had the highest BMI change of −0.76 kg/m^2^, followed by those that ate out between 4 and 6 times a month, −0.69 kg/m^2^, and those that ate out between 1 and 3 times a month, −0.10 kg/m^2^. There was a significant difference between eating out and BMI change (*p* = 0.036) (Table 6).

### 3.7. Physical Activity

Most of the cohort (Table 7) were ‘moderately inactive’ (32.3%), with some age group differences followed by ‘inactive’ (29%) who were all aged between 30–39. In all 4 measurement meetings, the women that were moderately active had the lowest BMI and the least active women had the highest BMI. For the majority of participants, activity levels did not change during the study.

### 3.8. Diet Diaries

The diet diaries reported a mix of the traditional Asian diet dishes and typical foods from the western diet which were mainly energy-dense takeaway foods (e.g., pizza, fried chicken, and chips) typically high in saturated fat, salt, and calories. Take-away consumption didn’t change during the intervention however the diet diaries showed increased consumption of salads and vegetables with meals.

**Figure 4:**
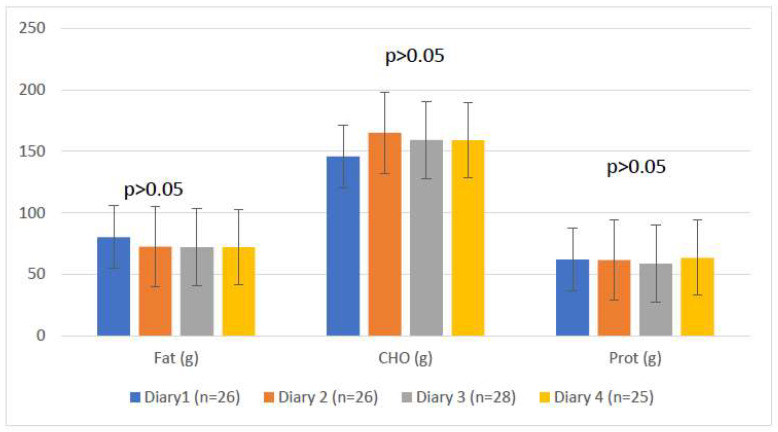
Macronutrient intake of the participants. Data are presented as mean and *p*-values obtained by independent *t*-test.

The mean energy intake of the participants by week as reported in the diaries was 1504.68 kcal. Mean reported energy intakes at baseline and week 16 were 1521.30 kcal and 15,011.66 kcal, respectively. The mean intake of fat decreased across the study (Figure 4).

The calculated energy intake was compared to the BMI of the participants; the reported energy intake reported was low for the individuals’ BMI suggesting underreporting. This was confirmed by calculating under/over-reporting. The evidence suggests the mean under-reporting across the study was 92.7% (90.3–96.8). Mean normal reporting was 7.3% (3.2–9.7). None of the participants over-reported intakes. The mean difference between diary reported intakes and actual intakes is −788.95 kcal, as calculated (using the method according to [34]).

### 3.9. Three Factor Eating Questionnaire

The mean dietary restraint (DR) score was 10.52 (SD 4.3), dietary disinhibition (DD) score was 8.47 (SD 4.7) and hunger (H) score was 6.87 (SD 4.7), respectively (Table 8). DR was lowest pre-intervention at 10.32, increased to the highest score at week 4 (10.71), and decreased both at week 8 and 16 with a final score of 10.45, higher than the initial score (Table 8). This could suggest that the tools helped the participants and made their self-control stronger.

DD followed the opposite pattern, starting with the highest score of 8.74 at pre-intervention, decreasing to 8.32 at week 4 and week 8 increasing at week 16 to 8.48, yet it was still a lower score than before the study started. This could suggest that as the participants felt they had more control that they also felt less likely to be tempted to eat more.

Reported hunger scores start with the mean score of 7.10, reduce to 6.58 (week 4), then reduce to 6.52 with the lowest score in week 8. However, this score increases again by week 16.

### 3.10. Portion Control Self-Efficacy

These questions centred on food portion control and influences. The most common answers to questions indicated the level of agreement with the statement using a Likert scale. The mean results are presented in Table 9. The results suggest that the participants have a strong belief in their own ability to perform a behaviour related to controlling the portion of food consumed and this did not change significantly during the intervention.

## 4. Discussion

Thirty-one first and second-generation overweight or obese migrant South Asian, Muslim women from the north of England at risk of diabetes and other NCDs completed a cross-over trial aimed at investigating the acceptability, usability, efficacy, effectiveness, and the likelihood of continued use of two sets of commercially available calibrated portion size tools with the aim to control portion size and aid weight management and/or weight loss. This represented 61% of those initially recruited and is a positive outcome for studies of this nature and over a longer period of time. We did not have to recruit the full 75 sample size estimated due to the lower-than-expected dropout rate enabling us to achieve the final sample size. This was in part achieved through the use of a digital social media (WhatsApp) group sharing pictures of meals and regular telephone reminders from locally recruited research assistants.

Overall, the findings suggest the use of the tools resulted in weight loss and anthropometric changes with a statistically significant reduction in both BMI and weight over the period of the study. These findings are comparable to other weight-loss interventions in the South Asian community. Rush et al. (2007) reported decreases in anthropometric measures in both men and women over a five-month diet and physical activity intervention in an Asian Indian community group [36]. Additionally, a pilot exercise and healthy eating group in North West England reported weight and BMI decrease in 13 overweight or obese Asian women [37]. The changes in weight were likely due to food-related changes (consuming more salad and vegetables and less energy-dense foods) as a result of participation in the intervention. Furthermore, a recent meta-analysis reports portion control tools marginally induced weight loss especially driven by calibrated tableware [38].

A large proportion (62%) of the participants reported very low or inactive levels of physical activity. This did not change for the majority of the participants for the duration of the intervention. Other literature reports that South Asians have lower levels of self-reported and actual physical activity than Europeans [39,40,41,42,43,44]. There is extensive literature that discusses the challenges and barriers to undertaking physical activity, including simple walking in this group [45,46,47,48,49]. Furthermore, quantitative evidence indicates South Asian women do not perform the recommended level of PA for health benefits [39,44,45]. Many of the women joining the study report, at the screening stage, similar challenges and have a preference to try dietary methods for losing weight. Furthermore, our findings support the literature and that despite being on an intervention aimed at weight reduction they did not change their level of activity for the duration of the intervention. Thus, we conclude the weight-related results observed are not influenced by any changes in physical activity during the same period.

Diet diaries were used to capture changes in dietary intake and its composition. There was no significant difference in energy intake across the intervention when diaries were assessed quantitatively. However, we observe lower energy intake ratios compared to the BMR. This suggests that there is a significant amount of under-reporting in this study (mean 97.3%), with the mean under-reporting being 788.41 kcal which is not unusual in dietary studies. This may in part be explained by the lower literacy levels in English in this group and their ability to quantify foods that could impact energy intake data. We observed that quantification was better with the use of the portion size estimation tools. However, more work around this aspect is warranted to be meaningful for assessing dietary intakes for epidemiological or other studies. Many of the women could not read or write in English and so were assisted by family members or an interpreter to complete the diaries. This may also influence under-reporting [50]. Furthermore, it is possible that participants modified their reported behaviour (food intakes) in response to their awareness of being observed (Hawthorne effect) [51].

The diet diaries were regarded as a burdensome method for this participant group. This is an acknowledged challenge for nutritional surveillance in this South Asian group. Alternative methods may be more appropriate, such as photographic food atlas or household measures to assist data collection [16]. Some foods, for example, chapati, used a set value, yet this food can be made in a number of sizes and weights and would therefore influence intake and nutritional intakes. Thus, additional visual methods to assist quantification would be useful for research in this group. In validation and comparison studies, food image-based portion size estimation tools were found to be more accurate than food models and household utensils [52]. An amended diary with pictures of the tools for the intervention study weeks may aid data collection. Furthermore, digital technology has been reported to be effective for portion size measurements and could be explored for use with this group as many of the women in the study group used the “WhatsApp” social media application to share pictures of their meals and this should be built upon further. This aspect also reportedly helps motivation and understanding of simple instructions [53] as was the case in our study.

Despite the above limitations, the diet diaries provided useful insight into the types of foods eaten by participants. The diet reported is a mix of the traditional Asian diet dishes and typical foods from the western diet. The latter were mainly energy-dense takeaway foods typically high in saturated fat, salt, and calories. These are common take-away foods in the UK [54]. Some of the densest concentrations of fast food outlets are found in England’s poorest and most deprived neighbourhoods, which are a characteristic of the study areas in this research [55]. This is an area to be addressed in future health education/promotion strategies with this community as a focus on environmental level strategies is warranted. However, whilst takeaway consumption did not change during the intervention the diet diaries showed an increased consumption of salads and vegetables with meals. This was emphasised in the instructions in relation to the Eatwell guide.

What is interesting is that for those women who did not eat out as often (consumed less non-home cooked food) the BMI change (decrease) is greater. This suggests that the dietary changes which occurred in this study are likely to have been in home-cooked foods. This also suggests that whilst food-level strategies for the reduction of portion size which focus on manufacturers and restaurants are important [18], individual-level strategies could have more impact and need to be further developed.

From the TFEQ results dietary restraint showed some improvement during the first stages of the intervention suggesting the participants had greater self-control, but this lessened during the 8 weeks where participants were on their own using both tools (T3). DD followed the opposite pattern, starting with the highest score at pre-intervention, decreasing at week 4 and week 8 before increasing slightly at week 16. This could suggest that as the participants progressed in the study, they felt they had more control and that they also felt less likely to be tempted to eat more.

Reported hunger scores showed a decrease until week 8 indicating the use of the tools/changes in diet assisted with better control over hunger. This could be consistent with the reported changes in types of food eaten and quantities, for example, more salads/vegetables consumed. However, again the scores increase by week 16. This may suggest that the participants stayed motivated during the early stages of the intervention possibly due to the contact with the researcher, compared to when they were encouraged to use both tools for a longer period of time. Future adaptations could include a telephone call to coach participants (rather than remind participants) during the periods of the intervention which may boost motivation, an approach that has been successful in other weight loss intervention studies [56].

The PCSE results suggest that the participants have a strong belief in their own ability to perform a behaviour related to portion control. This is checked and reinforced by the question “it would be easy for me to control the size of the portions that I eat at social events at home” which is articulated as a reverse question, but the results were in line with the other responses. However, despite translators being trained and terminology agreed upon, we cannot ignore participant understanding and interpretation of the question which may influence the answers.

Both portion control tools were deemed to be acceptable, usable, and effective for day-to-day use by the participants (mean score above >3.2 on a scale of 1–5). However, the crockery set scored higher in all the aforementioned factors, an indication that the women were able to integrate and use the tools into the type of foods they commonly eat and adaptable to the way in which most Asian foods are served namely, at the table from a common pot. Given this coupled with the nature of the amorphous foods, typical of a South Asian diet we may have expected the utensils to score higher on all these three factors. Qualitative feedback, not reported herein, suggest that the tools were useful in the kitchen for serving but not at a dining table due to their size.

Some of the portion size tools were deemed to be rather large portions e.g., the soup ladle which was used for lentil-based curries. This could have a negative effect on use. Hollands et al. report the effects of size on selection in that adults always chose the larger size when offered larger-sized portions, packages, or items of tableware than when offered smaller-sized versions with the size of this effect small to moderate [57]. Although our study focussed on self-served portions rather than those on offer it may be that different-sized utensils or crockery could help portion control for commonly consumed South Asian foods.

Our study explored the experiences of individuals with a BMI greater than 23 (as having an increased risk of co-morbidities such as diabetes), in their local environment. Although this was also a non-controlled environment, it represents a more realistic real-world context in which such tools may be used in a consistent manner. In our study, dietary and physical activity components were similar to recommendations for the general UK population provided through guidance at the first meeting. However, feedback from participants during the debrief session suggested more tailored recommendations for future interventions would be useful. This is supported by findings of a systematic review for interventions for South Asians with diabetes type 2 [17] and our own earlier work [11].

The self-directed nature of the intervention meant that minimal nutritionist input was provided. Our study has demonstrated that for this group simple advice and the visual and quantitative nature of the tools appears to be effective for controlling portion size and composition of the diet. To the best of our knowledge, this is the first study of its kind on free-living South Asian participants to use portion size estimation tools in this way. The results are promising for a wider-scale intervention based on portion assessment and control. Bhopal, Douglas, et al. (2014) report on a family-cluster randomised controlled trial in a clinical setting focussed on weight control and physical activity in South Asian individuals in the UK [58]. They report modest, medium-term changes in weight are achievable as a component of lifestyle-change strategies, which might control or prevent adiposity-related diseases. Our findings are comparable to those reported by others from a study using non-Asian participants recruited from a community weight loss programme and dietetic service, where a similar intervention was undertaken [32]. Weight loss achieved is reported to be −1.7 (SD 4.1) kg (*p* < 0.05), compared to our findings of −0.97 (SD 1.74) kg (*p* = 0.04). The authors also report that self-selected portion sizes increased for vegetables and decreased for chips and potatoes with both tools. Participants rated both tools as equally acceptable, easy to use, and with similar perceived effectiveness. The intervention effect of the researchers cannot be overlooked as a motivating factor. However, as contact was minimal (except for reminders of weigh-in times) this effect is reduced.

The efficacy, acceptability, applicability, and effectiveness of this study’s tools provide a basis for their use in future studies. However, since our results indicate the portion control utensils were less suitable for the types of foods commonly consumed, additional alternatives from the range of other commercially available tools could be explored. Key factors to consider when selecting a portion size estimation tool are the applicability to the commonly consumed foods by the target population and the use at the table for communities where serving occurs at the meal table. Furthermore, wider changes to the portion-size environment may be necessary to support individual strategies leading to portion control.

In addition to the issues of literacy discussed above, other limitations are worth noting. Challenges in measuring waist circumference in this group relate to the measurement of the umbilicus which is not reliable because sagging of abdominal skin occurs in very obese participants, and this affects the size and position of the umbilicus. This is particularly so in women who have had a number of pregnancies or those who may have lost weight previously, as observed in this study [59]. This was in part addressed by the training of research assistants and using the same researcher for each measure on the same participant.

Limitations of language barriers were in part addressed using trained bilingual Punjabi/Urdu) translators with back translations being used to enhance quality. However, despite this, the subtlety of the terminology used in the psychological questionnaires is not easily translatable and we had low education levels in our sample, and this could influence understanding and hence responses. The use of Cronbach’s alpha coefficient to check intelligibility is recommended for future use. However, given the questionnaire had been reliable for use in South Asian groups by previous researchers [60] we did not undertake this step in our study. Validation of the translated version would also be useful for future work.

## 5. Conclusions

It is widely acknowledged that obesity needs to be reduced in this South Asian group, particularly since the pandemic where the link between lower BMIs to mortality associated with COVID-19 was recently reported in this ethnic group [10]. Our study has shown modest weight loss and increased ability to regulate portion size (particularly early on) in our study sample. Designing successful interventions for use in hard-to-reach ethnic minority communities (who often stay at home) is challenging. The co-creation of the study design is a positive aspect and should be continued for future research as it also enhanced recruitment. This was assisted by the use of researchers from the community settings who have local knowledge and could disseminate information about the intervention and assist in recruiting participants. Finally, our sample had low activity levels which did not change during the study and therefore highlights the importance of weight reduction through dietary approaches for addressing overweight and obesity in this ethnic group. Physical activity should however continue to be encouraged despite its reported barriers.

This study has uniquely generated evidence that portion control tools including guided and calibrated tableware are perceived as acceptable and easy to use by South Asian women who wanted to maintain (not get heavier) or lose weight. The approach has the potential to achieve modest weight loss with minimal health professional input. The findings have generalisability to a wider South Asian group The portion control tools tested herein provide a simple and relatively inexpensive strategy that could be further developed and implemented alongside other strategies as part of weight management interventions. It also has scope for use with South Asians living with comorbidities although not specifically tested in this study. Further research over a longer period is needed to explore whether the approach results in weight loss and maintenance in the longer term and explore what additional support may be necessary. This could include the use of digital technologies. This paper adds to the limited body of evidence on dietary interventions to enable weight loss within the South Asian female population.

## Figures and Tables

**Table 1:** Baseline Characteristics’ for the Participants (*n* = 31).

Age (Years):	*n*	%
18–29	6	19
30–39	10	32
40–49	10	32
50+	5	16
Ethnicity:		
Asian	20	64.5
Asian British	11	35.5
Education:		
Primary	2	7
High school	18	58
University	6	19
Other	5	16
Married	26	83.9
Had children	25	80.6
Mean number of children	2.7	-
SD number of children	1.6	-

Numbers (*n*) and percentages (%), means and standard deviation (SD).

**Table 2:** Breakfast and homemade food consumption pattern.

	Number of Times a Week Breakfast Is Consumed		Number of Days a Week Homemade Food Is Consumed	
	*n*	%	*n*	%
<2	3	9.7	2	6.5
2–5	2	6.5	3	9.7
4–6	3	9.7	18	58.1
7	23	74.2	8	25.8
Total	31	100.0	31	100.0

**Table 3:** Acceptance, ease of use and effectiveness score by tool.

	Utensils Set		Crockery Set		Ppt Chose from both Sets ^1^	
	Mean	SD	Mean	SD	Mean	SD
Mean acceptance score (1–5)	3.63	0.46	4.04	0.69	-	-
Mean ease of use score (1–5)	3.39	0.68	4.04	0.73	-	-
Mean effectiveness score (1–5)	3.28	0.83	4.13	0.67	-	-
VAS score *	6.56	2.1	7.73	1.8	7.00	2.47

Observed covariance matrices of the dependent variables are equal across groups, therefore results presented as means and SD. * Likelihood of continued use (1–100 mm). ^1.^ For the final stage of the intervention the participants were asked to use their preferred tools from both sets.

**Table 4:** Common self-reported changes in preparation, serving or consumption of food groups (scores from Q1, Q2, and Q3).

	Utensils	Crockery
**Increased**	Vegetables, salad	Salad
**Decreased**	Fruit, bread, breakfast cereal, pasta, milk/yoghurt, savoury snacks, confectionery, butter, cooking oil	Breakfast cereal, milk/yoghurt cooking oil
**Not changed**	Rice, potatoes, meat, pulses, cheese, meat/fish,	Vegetables, rice, pasta, potatoes, chips/roast potatoes, pulses
**Portion size tools not used for**	Chips/roast potatoes,	Fruit, bread, cheese, meat/fish, savoury snacks, confect, butter, measuring cooking oil

**Table 5:** Mean SD and range of weight, BMI, and waist circumference at baseline and change.

Weight (kg)	Baseline	Week 16	Change
Mean	79.86	78.89	−0.97 (*p* = 0.004)
SD	14.11	13.38	1.74
Range (Min–Max)	57.60–108.6	57.60–105.30	
**BMI (kg/m^2^)**			
Mean	31.61	31.26	−0.35 (*p* = 0.007)
SD	4.98	4.75	0.68
Range Min–Max	23.48–40.61	23.48–39.05	
**Waist Circumference (cm)**			
Mean	102.93	101.41	−1.52 (*p* = 0.049)
SD	12.99	51.40	4.72
Range Min–Max	78.80–137.00	78.60–130.00	

**Table 6:** Eating out and BMI change.

Eating out (Times Per Month)	% (*n*)	Mean	SD	Min	Max	*p*
<1	29.0 (9)	−0.7556	0.74179	−1.68	0.08	0.036
1–3	61.3 (19)	−0.1047	0.56544	−127	1.35	
4–6	9.7 (3)	−0.6867	0.62429	−1.22	0.00	

**Table 7:** Mean activity level by age and BMI and by meeting number Total (*n* = 31).

							Meeting No.			
Activity Level			Age (yrs.)				1	2	3	4
	*n*	%	18–29	30–39	40–49	50+	BMI (kg/m^2^)			
Active	5	16.1	1	1	3	0	31.02	31.06	30.85	30.51
Moderately active	7	22.6	3	1	3	0	30.08	29.98	29.85	29.79
Moderately inactive	10	32.3	1	5	2	2	31.13	31.09	29.84	30.69
Inactive	9	29	1	3	2	3	33.73	33.68	30.93	33.44

**Table 8:** Three Factor Eating Questionnaire Results.

	Week						T1	T2	T3	T4
	1	4	8	16	Mean	SD	(Q2 − Q1)	(Q3 − Q2)	(Q4 − Q3)	(Q4 − Q1)
Dietary Restraint (max score 21)	10.32	10.71	10.61	10.45	10.52	4.3	0.39	−0.10	−0.16	0.13
Dietary Disinhibition (max score 16)	8.74	8.32	8.32	8.48	8.47	4.7	−0.42	0.0	−0.42	−0.27
Hunger (max score 14)	7.10	6.58	6.52	7.29	6.87	4.7	−0.52	−0.06	0.19	0.19

**Table 9:** Mean scores from Portion Control Self-Efficacy scale (*n* = 31).

	Baseline	Tool 1	Tool 2	Both
Opinions:	PCSE T_0_	PCSE T_1_	PCSE T_2_	PCSE T_3_
I believe I can eat standard food portions when served portions that are too large.	3.48 ± 1.092	3.32 ± 1.013	3.52 ± 1.029	3.45 ± 1.060
I can handle eating the right food portions no matter what comes my way.	3.26 ± 1.125	3.03 ± 1.169	3.39 ± 1.054	3.29 ± 1.160
I feel confident that I can leave food on my plate if I think a serving size is too large.	3.42 ± 1.177	3.35 ± 1.253	3.35 ± 1.170	3.23 ± 1.175
When eating with others, they influence how much I eat.	3.23 ± 1.283	3.26 ± 1.264	3.32 ± 1.166	3.35 ± 1.142
It would be easy for me to control the size of the portions that I eat at social events home.	3.16 ± 1.003	3.16 ± 1.036	3.23 ± 1.117	3.13 ± 0.957
I don’t know if I can control the size of the portions that I eat at home.	3.03 ± 1.251	2.94 ± 1.237	3.06 ± 1.063	3.35 ± 1.082
I am confident that I can control the size of the portions that I eat when at home with others (leave blank if not applicable to you).	3.29 ± 1.131	3.35 ± 1.170	3.35 ± 0.985	3.29 ± 0.938
I am confident I can judge whether a serving is appropriate when eating at home with others (leave blank if not applicable to you).	3.35 ± 1.018	3.58 ± 1.089	3.55 ± 0.925	3.52 ± 1.029

## Data Availability

Data can be obtained by requesting in writing to the corresponding author.

## Supplementary Materials

The following supporting information can be downloaded at: https://www.mdpi.com/article/10.3390/ijerph19137714/s1, File S1a: questionnaires 1 and 2 used at T0, T1; File S1b: questionnaire 3 used at T2; File S2a: (Healthy Steps (Jokari) instructions); File S2b: Precise Portions instructions; File S3: Analysis of Questionnaires 1, 2 and 3.

## References

[1] Office for National Statistics (ONS) 2011 Census Analysis: Ethnicity and Religion of the Non-UK Born Population in England and Wales: 2011. https://www.ons.gov.uk/peoplepopulationandcommunity/culturalidentity/ethnicity/articles/2011censusanalysisethnicityandreligionofthenonukbornpopulationinenglandandwales/2015-06-18.

[2] Government UK (2021). Active Lives Adult Survey Official Statistics. https://www.ethnicity-facts-figures.service.gov.uk/health/diet-and-exercise/overweight-adults/latest.

[3] Sproston K., Mindell J.S. (2006). Health Survey for England 2004.

[4] Gujral U.P., Pradeepa R., Weber M.B., Narayan K.M., Mohan V. (2013). Type 2 diabetes in South Asians: Similarities and differences with white Caucasian and other populations. Ann. N. Y. Acad. Sci..

[5] Meeks K.A., Freitas-da-Silva D., Adeyemo A., Beune E.J., Modesti P.A., Stronks K., Zafarmand M.H., Agyemang C. (2016). Disparities in type 2 diabetes prevalence among ethnic minority groups resident in Europe: A systematic review and meta-analysis. Intern. Emerg. Med..

[6] Meeks K.A., Stronks K., Beune E.J., Adeyemo A., Henneman P., Mannens M.M., Nicolaou M., Peters R.J., Rotimi C.N., Snijder M.B. (2015). Prevalence of type 2 diabetes and its association with measures of body composition among African residents in the Netherlands—The HELIUS study. Diabetes Res. Clin. Pract..

[7] Franz M.J., Boucher J.L., Rutten-Ramos S., VanWormer J.J. (2015). Lifestyle weight-loss intervention outcomes in overweight and obese adults with type 2 diabetes: A systematic review and meta-analysis of randomized clinical trials. J. Acad. Nutr. Diet..

[8] National Institute of Health and Care Excellence (NICE) Type 2 Diabetes in Adults: Management. NICE Guideline [NG28]. https://www.nice.org.uk/guidance/ng28/resources/type-2-diabetes-in-adults-pdf-2830067254213.

[9] Leung G., Stanner S. (2011). Diets of minority ethnic groups in the UK: Influence on chronic disease risk and implications for prevention. Nutr. Bull..

[10] Yates T., Summerfield A., Razieh C., Banerjee A., Chudasama Y., Davies M.J., Gillies C., Islam N., Lawson C., Mirkes E. (2022). A population-based cohort study of obesity, ethnicity and COVID-19 mortality in 12.6 million adults in England. Nat. Commun..

[11] Ludwig A.F., Cox P., Ellahi B. (2011). Social and cultural construction of obesity among Pakistani Muslim women in North West England. Public Health Nutr..

[12] Choudhury S.M., Brophy S., Williams R. (2009). Understanding and beliefs of diabetes in the UK Bangladeshi population. Diabet Med..

[13] Ledikwe J.H., Ello-Martin J.A., Rolls B.J. (2005). Portion sizes and the obesity epidemic. J. Nutr..

[14] Kesman R.L., Ebbert J.O., Harris K.I., Schroeder D.R. (2011). Portion control for the treatment of obesity in the primary care setting. BMC Res. Notes.

[15] Pedersen S.D., Kang J., Kline G.A. (2007). Portion control plate for weight loss in obese patients with type 2 diabetes mellitus: A controlled clinical trial. Arch. Intern. Med..

[16] Almiron-Roig E., Aitken A., Galloway C., Ellahi B. (2017). Dietary assessment in minority ethnic groups: A systematic review of instruments for portion-size estimation in the United Kingdom. Nutr. Rev..

[17] Muilwijk M., Nicolaou M., Qureshi S.A., Celis-Morales C., Gill J.M.R., Sheikh A., Sattar N., Beune E., Jenum A.K., Stronks K. (2018). Dietary and physical activity recommendations to prevent type 2 diabetes in South Asian adults: A systematic review. PLoS ONE.

[18] Almiron-Roig E., Forde C.G., Hollands G.J., Vargas M.A., Brunstrom J.M. (2019). A review of evidence supporting current strategies, challenges and opportunities to reduce portion sizes. Nutr. Rev..

[19] Liu J.J., Davidson E., Bhopal R., White M., Johnson M., Netto G., Sheikh A. (2016). Adapting health promotion interventions for ethnic minority groups: A qualitative study. Health Promot. Int..

[20] National Institute of Health and Care Excellence (NICE) BMI: Preventing Ill Health and Premature Death in Black, Asian and Other Minority Ethnic Groups. https://www.nice.org.uk/guidance/ph46/history.

[21] Aitken A., Almiron-Roig E., Ellahi B. (2015). Preference and perceived useability of portion size tools in migrant South Asian Women. Ann. Nutr. Metab..

[22] Harris J.E., Raynor H.A. (2017). Crossover Designs in Nutrition and Dietetics Research. J. Acad. Nutr. Diet..

[23] Center for Disease Control and Prevention (CDC) (2007). Anthropometry Procedures Manual. https://docs.google.com/viewer?url=https%3A%2F%2Fwww.cdc.gov%2Fnchs%2Fdata%2Fnhanes%2Fnhanes_07_08%2Fmanual_an.pdf.

[24] Stunkard A.J., Messick S. (1985). The three-factor eating questionnaire to measure dietary restraint, disinhibition and hunger. J. Psychosom. Res..

[25] Fast L.C., Harman J.J., Maertens J.A., Burnette J.L., Dreith F. (2015). Creating a measure of portion control self-efficacy. Eat Behav..

[26] Bull F.C., Maslin T.S., Armstrong T. (2009). Global physical activity questionnaire (GPAQ): Nine country reliability and validity study. J. Phys. Act Health.

[27] Public Health England (PHE) The Eatwell Guide. https://www.nhs.uk/live-well/eat-well/the-eatwell-guide/.

[28] HealthyStepsTM Healthy Steps Portion Control Serving Set. www.myhealthysteps.com.

[29] Food and Drug Administration Centre for Food Safety and Applied Nutrition (FDA) A Food Labelling Guide Guidence for Industry. https://www.fda.gov/downloads/food/guidance%20complianceregulatoryinformation/%20guidancedocuments/foodlabelingnutrition/foodlabelingguide/ucm265446.pdf.

[30] Precise Portions Precise Portions Nutrition Control System. https://www.preciseportions.com/.

[31] Almiron Roig E., Vaughan D., Jebb S.A. (2012). Acceptance of Portion Size Tools Amongst Overweight Individuals.

[32] Almiron-Roig E., Dominguez A., Vaughan D., Solis-Trapala I., Jebb S.A. (2016). Acceptability and potential effectiveness of commercial portion control tools amongst people with obesity. Br. J. Nutr..

[33] Schofield N.W. (1985). Predicting basal metabolic rate, new standards and review of previous work. Hum. Nutr. Clin. Nutr..

[34] Johansson L., Solvoll K., Bjorneboe G.E., Drevon C.A. (1998). Under- and overreporting of energy intake related to weight status and lifestyle in a nationwide sample. Am. J. Clin. Nutr..

[35] National Institute of Health and Care Excellence (NICE) Preventing Type 2 Diabetes Risk: Identification and Interventions for Individuals at High Risk. https://www.nice.org.uk/guidance/ph38.

[36] Rush E.C., Chandu V., Plank L.D. (2007). Reduction of abdominal fat and chronic disease factors by lifestyle change in migrant Asian Indians older than 50 years. Asia Pac. J. Clin. Nutr..

[37] Williams J., Sultan M. (1999). Evaluation of an Asian women’s healthy eating and exercise group. J. Hum. Nutr. Diet..

[38] Vargas-Alvarez M.A., Navas-Carretero S., Palla L., Martinez J.A., Almiron-Roig E. (2021). Impacct of portion contol tools on por-tion size awareness, choice and intake: Stystematic review and Meta-Analysis. Nutrients.

[39] Fischbacher C.M., Hunt S., Alexander L. (2004). How physically active are South Asians in the United Kingdom? A literature review. J. Public Health.

[40] Afaq S., Kooner A.S., Loh M., Kooner J.S., Chambers J.C. (2019). Contribution of lower physical activity levels to higher risk of insulin resistance and associated metabolic disturbances in South Asians compared to Europeans. PLoS ONE.

[41] Pomerleau J., McKeigue P.M., Chaturvedi N. (1999). Factors associated with obesity in South Asian, Afro-Caribbean and European women. International journal of obesity and related metabolic disorders. J. Int. Assoc. Study Obes..

[42] Hayes L., White M., Unwin N., Bhopal R., Fischbacher C., Harland J., Alberti K.G. (2002). Patterns of physical activity and relationship with risk markers for cardiovascular disease and diabetes in Indian, Pakistani, Bangladeshi and European adults in a UK population. J. Public Health Med..

[43] Williams E.D., Stamatakis E., Chandola T., Hamer M. (2011). Assessment of physical activity levels in South Asians in the UK: Findings from the Health Survey for England. J. Epidemiol. Community Health.

[44] Biddle G.J.H., Edwardson C.L., Rowlands A.V., Davies M.J., Bodicoat D.H., Hardeman W., Eborall H., Sutton S., Griffin S., Khunti K. (2019). Differences in objectively measured physical activity and sedentary behaviour between white Europeans and south Asians recruited from primary care: Cross-sectional analysis of the PROPELS trial. BMC Public Health.

[45] Bhatnagar P., Shaw A., Foster C. (2015). Generational differences in the physical activity of UK South Asians: A systematic review. Int. J. Behav. Nutr. Phys. Act..

[46] Darr A., Astin F., Atkin K. (2008). Causal attributions, lifestyle change, and coronary heart disease: Illness beliefs of patients of South Asian and European origin living in the United Kingdom. Heart Lung.

[47] Horne M., Skelton D., Speed S., Todd C. (2010). The influence of primary health care professionals in encouraging exercise and physical activity uptake among White and South Asian older adults: Experiences of young older adults. Patient Educ. Couns..

[48] Lawton J., Ahmad N., Hanna L., Douglas M., Hallowell N. (2006). ‘I can’t do any serious exercise’: Barriers to physical activity amongst people of Pakistani and Indian origin with Type 2 diabetes. Health Educ. Res..

[49] McKenna J., Ludwig A.F. (2008). Osteoporotic Caucasian and South Asian women: A qualitative study of general practitioners’ support. J. R. Soc. Promot. Health.

[50] Macdiarmid J., Blundell J. (1998). Assessing dietary intake: Who, what and why of under-reporting. Nutr. Res. Rev..

[51] Payne G., Payne J. (2004). The hawthorne effect. Sage Key Concepts: Key Concepts in Social Research.

[52] Amoutzopoulos B., Page P., Roberts C., Roe M., Cade J., Steer T., Baker R., Hawes T., Galloway C., Yu D. (2020). Portion size estimation in dietary assessment: A systematic review of existing tools, their strengths and limitations. Nutr. Rev..

[53] Yang Y., Jia W., Bucher T., Zhang H., Sun M. (2019). Image-based food portion size estimation using a smartphone without a fiducial marker. Public Health Nutr..

[54] GGouk A. (2016). What’s the most popular takeaway food where you live?. Manchester Evening News.

[55] Public Health England (PHE) England’s Poorest Areas Are Fast Food Hotspots. New Figures from PHE Show Higher Concentrations of Fast Food Outlets in England’s Most Deprived Communities. https://www.gov.uk/government/news/englands-poorest-areas-are-fast-food-hotspots.

[56] Tapsell L.C., Lonergan M., Batterham M.J., Neale E.P., Martin A., Thorne R., Deane F., Peoples G. (2017). Effect of interdisciplinary care on weight loss: A randomised controlled trial. BMJ Open.

[57] Hollands G.J., Shemilt I., Marteau T.M., Jebb S.A., Lewis H.B., Wei Y., Higgins J.P., Ogilvie D. (2015). Portion, package or tableware size for changing selection and consumption of food, alcohol and tobacco. Cochrane Database Syst. Rev..

[58] Bhopal R.S., Douglas A., Wallia S., Forbes J.F., Lean M.E., Gill J.M., McKnight J.A., Sattar N., Sheikh A., Wild S.H. (2014). Effect of a lifestyle intervention on weight change in south Asian individuals in the UK at high risk of type 2 diabetes: A family-cluster randomised controlled trial. Lancet Diabetes Endocrinol..

[59] Sattar N., Lean L. (2007). ABC of Obesity.

[60] De Lauzon B., Romon M., Deschamps V., Lafay L., Borys J.M., Karlsson J., Ducimetiere P., Charles M.A., Fleurbaix Laventie Ville Sante Study G. (2004). The Three-Factor Eating Questionnaire-R18 is able to distinguish among different eating patterns in a general population. J. Nutr..

