# Supplementary material for: Acceptability, Usability and Weight Loss Outcomes in a Randomized Cross-Over Study of Commercially Available Portion Size Tools in an Overweight South Asian Community
Source: Int J Environ Res Public Health. 2022 Jun 23;19(13):7714. doi: 10.3390/ijerph19137714 (PMC9266172; doi:10.3390/ijerph19137714)
Supplement: Supplementary file 1 [file ijerph-19-07714-s001.zip › ijerph-1747504-supplementary.pdf]

Supplementary information summary

S1a: questionnaires 1 1nd 2 used at T0, T1 (S1a)

S1b: questionnaire 3 used at T2

S2a: (Healthy Steps (Jokari) instructions)

S2b: Precise Portions instructions

S3: Analysis of Questionnaires 1,2 and 3

Researcher/Volunteer ID number:

S1a: Study Questionnaire 1 & 2 (T0 and T1)

Portion size tools for weight management in South Asian women

To be completed by investigator

Volunteer ID number: ______________________

Date when this questionnaire was given out: ______________________

Study stage recently completed (please tick one only. Indicate last day of the 4 weeks trial period):

☐ First tool trial Completed on: ……..

Instructions for completing this questionnaire

- The following questionnaire will aid the investigators of this study to learn more about your experience with the portion size tools you have tried.
- There are no right or wrong answers. Please be as honest as you can.
- The questionnaire consists of 10 questions, some of which have multiple parts. It should take you no more than 15 min to complete it.
- Once completed, **please return to the investigator on your next visit.**

QUESTIONNAIRE

Dates when you used the tool: First day: .... Last day: ....

Question 1. Which tool have you just tried out? (please tick one):

☐ Crockery set ☐Serving spoon set

Question 2. Over the last 2 weeks, how many days a week did you use this tool, or parts of it, on average?

☐	Never or once in the 4 weeks
☐	1-2 days a week
☐	3-5 days a week
☐	Most days of the week (6-7 days)
☐	Used it only for a few days at the beginning or at the end

Question 3. For which meals did you tend to use this tool (or parts of the tool)? (Tick all that apply)

☐ Breakfast ☐ Lunch ☐ Evening meal ☐ Snacks/drinks

Question 4. The following set of statements refers to your experience with *the specific tool or tool set* that you have just used (indicated in response to Question 1 above). Please indicate your level of agreement with each statement (please circle one number only):

	Strongly disagree				Strongly agree
a. I liked the look of the tool	**1**	**2**	**3**	**4**	**5**
b. This tool fits with the rest of my kitchenware	**1**	**2**	**3**	**4**	**5**
c. This tool fits with my family/home life	**1**	**2**	**3**	**4**	**5**
d. I felt embarrassed using this tool at home/ with others	**1**	**2**	**3**	**4**	**5**

e. This tool was easy to use	**1**	**2**	**3**	**4**	**5**
f. This tool was resistant to wear and tear	**1**	**2**	**3**	**4**	**5**
g. The written instructions provided with this tool were clear	**1**	**2**	**3**	**4**	**5**
h. Compared with other tools I have seen/tried, this tool was more convenient to use. (If you haven't used other tools before tick here ☐)	**1**	**2**	**3**	**4**	**5**
i. This tool helped me learn what the appropriate portion size for particular foods is (e.g. for juice, pasta, or cereal)	**1**	**2**	**3**	**4**	**5**
j. I used the tool to measure foods I have never measured before	**1**	**2**	**3**	**4**	**5**
k. In general, once I used this tool for a particular food, I continued to use it for that food	**1**	**2**	**3**	**4**	**5**
l. Overall, this tool helped me meet my current dietary goals	**1**	**2**	**3**	**4**	**5**

Question 5. Was there any specific part or aspect of this tool that you found more useful? If so, please tell us which one(s) and why.

Question 6. We would like to ask you about how you used this tool. Please tell us if you think you have modified your usual portions when you have used the tool to prepare, serve or consume the following foods for yourself.

**Food**	**As a result of using the tool I have…**<br>(Please tick one box only for each food group)
Vegetables (cooked)<br>☐ *Did not consume*	☐ Increased size of serving (Increased)<br>☐ Decreased size of serving (Decreased)<br>☐ Have not changed the size of serving (Not changed)<br>☐ Did not use tool for these foods
Salad, raw vegetables<br>☐ *Did not consume*	☐ Increased<br>☐ Decreased<br>☐ Not changed<br>☐ Did not use tool for these foods
Fruits (excluding fruit juice and smoothies)<br>☐ *Did not consume*	☐ Increased<br>☐ Decreased<br>☐ Not changed<br>☐ Did not use tool for fruits
Bread<br>☐ *Did not consume*	☐ Increased<br>☐ Decreased<br>☐ Not changed<br>☐ Did not use tool for bread
Rice and other grains<br>☐ *Did not consume*	☐ Increased<br>☐ Decreased<br>☐ Not changed<br>☐ Did not use tool for these foods
Breakfast cereals<br>☐ *Did not consume*	☐ Increased<br>☐ Decreased<br>☐ Not changed<br>☐ Did not use tool for breakfast cereals
Pasta<br>☐ *Did not consume*	☐ Increased<br>☐ Decreased<br>☐ Not changed<br>☐ Did not use tool for pasta
Potatoes (boiled or mashed)<br>☐ *Did not consume*	☐ Increased<br>☐ Decreased<br>☐ Not changed<br>☐ Did not use tool for this food

Chips, roast/wedge potatoes<br>☐ *Did not consume*	☐ Increased<br>☐ Decreased<br>☐ Not changed<br>☐ Did not use tool for these foods
Milk, yoghurt and milk-based drinks, including non-dairy alternatives<br>☐ *Did not consume*	☐ Increased<br>☐ Decreased<br>☐ Not changed<br>☐ Did not use tool for these foods
Cheese<br>☐ *Did not consume*	☐ Increased<br>☐ Decreased<br>☐ Not changed<br>☐ Did not use tool for cheese
Meat, fish and meat or fish-based dishes (e.g. meat or fish pies)<br>☐ *Did not consume*	☐ Increased<br>☐ Decreased<br>☐ Not changed<br>☐ Did not use tool for these foods
Pulses, egg-based dishes; meat-free foods (e.g. vegetarian options)<br>☐ *Did not consume*	☐ Increased<br>☐ Decreased<br>☐ Not changed<br>☐ Did not use tool for these foods
Savoury snacks (e.g. crisps; nuts)<br>☐ *Did not consume*	☐ Increased<br>☐ Decreased<br>☐ Not changed<br>☐ Did not use tool for savoury snacks
Confectionary, cake, desserts<br>☐ *Did not consume*	☐ Increased<br>☐ Decreased<br>☐ Not changed<br>☐ Did not use tool for these foods
Butter; margarine<br>☐ *Did not consume*	☐ Increased<br>☐ Decreased<br>☐ Not changed<br>☐ Did not use tool for this food
Cooking oil, salad dressings<br>☐ *Did not consume*	☐ Increased<br>☐ Decreased<br>☐ Not changed<br>☐ Did not use tool for these foods

Question 7. There might have been times when you think you could have used the tool provided but you didn't use it. Did this happen to you? If so, please tell us when this was and why (please note, there are no right or wrong answers here).

Question 8. Please indicate on the scale below *how likely it would be that you continue to use this tool* in the long-term (beyond 4 weeks) as part of your dietary plans, if the tool was available to you (please draw a vertical line on the appropriate point).

Very Extremely

Question 9. Please tell us what you liked and did not like about using this tool in the following situations.

a) When portioning or serving foods for yourself

I liked:
I did not like:

b) Using the tools for other purposes, e.g. servings others in your family; for recipe preparation; serving foods during social occasions, etc.

I liked:
I did not like:

☐ Tick here if you only used the tools for serving foods for yourself.

Question 10. In your opinion, what would help to make this tool(s) more acceptable and/or useful in helping you meet your current dietary goals?

Finally, you may use the space on the next page for anything else you wish to tell us about your experience with this particular set of portion size tools.

This is the end of questionnaire.

THANK YOU VERY MUCH for your contribution.

Please return to your researcher at your next visit.

If you have any questions regarding this questionnaire or would like further clarification on how to fill it in please contact Amanda Aitken: a.aitken@chester.ac.uk or Professor Basma Ellahi – Faculty of Health and Social Care, University of Chester. Email address: b.ellahi@chester.ac.uk Telephone: 01244 512 177

Researcher/Volunteer ID number:

S1b: Study Questionnaire 3 (T2)

Portion size tools for weight management in South Asian women

To be completed by investigator

Volunteer ID number: ______________________

Date when this questionnaire was given out: ______________________

Study stage recently completed (please tick one only. Indicate last day of the 8 weeks trial period):

☐ both tools trial Completed on: ……..

Instructions for completing this questionnaire

- The following questionnaire will aid the investigators of this study to learn more about your experience with the portion size tools you have tried.
- There are no right or wrong answers. Please be as honest as you can.
- The questionnaire consists of 10 questions, some of which have multiple parts. It should take you no more than 15 min to complete it.
- Once completed, **please return to the investigator on your next visit.**

QUESTIONNAIRE

Dates when you used the tool: First day: .... Last day: ....

Question 1. Which tools did you use)? (Tick all that apply)

Crockery set

☐ Plate with writing ☐ plate without writing ☐ bowl

Serving spoon & measures set

☐ ladle ☐ pasta server ☐ starch server ☐ cereal scoop ☐ cheese grater

Question 2. Over the last 8 weeks, how many days a week did you use this tool, or parts of it, on average?

☐	Never or once in the 4 weeks
☐	1-2 days a week
☐	3-5 days a week
☐	Most days of the week (6-7 days)
☐	Used it only for a few days at the beginning or at the end

Question 3. For which meals did you tend to use this tool (or parts of the tool)? (Tick all that apply)

☐ Breakfast ☐ Lunch ☐ Evening meal ☐ Snacks/drinks

Question 4. The following set of statements refers to your experience with *the specific tool or tool set* that you have just used (indicated in response to Question 1 above). Please indicate your level of agreement with each statement (please circle one number only):

	Strongly disagree				Strongly agree
m. I liked the look of the tool	**1**	**2**	**3**	**4**	**5**
n. This tool fits with the rest of my kitchenware	**1**	**2**	**3**	**4**	**5**
o. This tool fits with my family/home life	**1**	**2**	**3**	**4**	**5**

p. I felt embarrassed using this tool at home/ with others	**1**	**2**	**3**	**4**	**5**
q. This tool was easy to use	**1**	**2**	**3**	**4**	**5**
r. This tool was resistant to wear and tear	**1**	**2**	**3**	**4**	**5**
s. This tool helped me learn what the appropriate portion size for particular foods is (e.g. for cheese, pasta, or cereal)	**1**	**2**	**3**	**4**	**5**
t. I used the tool to measure foods I have never measured before	**1**	**2**	**3**	**4**	**5**
u. In general, once I used this tool for a particular food, I continued to use it for that food	**1**	**2**	**3**	**4**	**5**
v. Overall, this tool helped me meet my current dietary goals	**1**	**2**	**3**	**4**	**5**

Question 5. Was there any specific part or aspect of this tool that you found more useful? If so, please tell us which one(s) and why.

Crockery set

☐ Plate with writing

Why____

☐ Plate without writing

Why____

☐ Bowl

Why____

Serving spoon & measures set

☐ ladle

Why____

☐ Pasta server

Why____

☐ Starch server

Why____

☐ Cereal scoop

Why____

☐ Cheese grater

Why____

Question 6. There might have been times when you think you could have used the tool provided but you didn't use it. Did this happen to you? If so, please tell us when this was and why (please note, there are no right or wrong answers here).

Question 7. Please indicate on the scale below *how likely it would be that you continue to use this tool* in the long-term (beyond 8 weeks) as part of your dietary plans, if the tool was available to you (please draw a vertical line on the appropriate point).

|———————————————————————|

Very   Extremely

Question 8. Please tell us what you liked and did not like about using this tool in the following situations.

c) When portioning or serving foods for yourself

I liked:<br><br><br>I did not like:

d) Using the tools for other purposes, e.g. servings others in your family; for recipe preparation; serving foods during social occasions, etc.

I liked:

I did not like:

☐ Tick here if you only used the tools for serving foods for yourself.

Question 9. In your opinion, what would help to make this tool(s) more acceptable and/or useful in helping you meet your current dietary goals?

Question 10. We would like to ask you about how you used this tool. Please tell us if you think you have modified your usual portions when you have used the tool to prepare, serve or consume the following foods for yourself.

**Food**	**As a result of using the tools for 8 weeks I have…** (Please tick one box only for each food group)
Vegetables (cooked)<br>☐ *Did not consume*	☐ Increased size of serving (Increased)<br>☐ Decreased size of serving (Decreased)<br>☐ Have not changed the size of serving (Not changed)<br>☐ Did not use tool for these foods
Salad, raw vegetables<br>☐ *Did not consume*	☐ Increased<br>☐ Decreased<br>☐ Not changed<br>☐ Did not use tool for these foods
Fruits (excluding fruit juice and smoothies)<br>☐ *Did not consume*	☐ Increased<br>☐ Decreased<br>☐ Not changed<br>☐ Did not use tool for fruits
Bread<br>☐ *Did not consume*	☐ Increased<br>☐ Decreased<br>☐ Not changed<br>☐ Did not use tool for bread
Rice and other grains<br>☐ *Did not consume*	☐ Increased<br>☐ Decreased<br>☐ Not changed<br>☐ Did not use tool for these foods
Breakfast cereals<br>☐ *Did not consume*	☐ Increased<br>☐ Decreased

	☐ Not changed<br>☐ Did not use tool for breakfast cereals
Pasta<br>☐ *Did not consume*	☐ Increased<br>☐ Decreased<br>☐ Not changed<br>☐ Did not use tool for pasta
Potatoes (boiled or mashed)<br>☐ *Did not consume*	☐ Increased<br>☐ Decreased<br>☐ Not changed<br>☐ Did not use tool for this food
Chips, roast/wedge potatoes<br>☐ *Did not consume*	☐ Increased<br>☐ Decreased<br>☐ Not changed<br>☐ Did not use tool for these foods
Milk, yoghurt and milk-based drinks, including non-dairy alternatives<br>☐ *Did not consume*	☐ Increased<br>☐ Decreased<br>☐ Not changed<br>☐ Did not use tool for these foods
Cheese<br>☐ *Did not consume*	☐ Increased<br>☐ Decreased<br>☐ Not changed<br>☐ Did not use tool for cheese
Meat, fish and meat or fish-based dishes (e.g. meat or fish pies)<br>☐ *Did not consume*	☐ Increased<br>☐ Decreased<br>☐ Not changed<br>☐ Did not use tool for these foods
Pulses, egg-based dishes; meat-free foods (e.g. vegetarian options)<br>☐ *Did not consume*	☐ Increased<br>☐ Decreased<br>☐ Not changed<br>☐ Did not use tool for these foods
Savoury snacks (e.g. crisps; nuts)<br>☐ *Did not consume*	☐ Increased<br>☐ Decreased<br>☐ Not changed<br>☐ Did not use tool for savoury snacks
Confectionary, cake, desserts<br>☐ *Did not consume*	☐ Increased<br>☐ Decreased<br>☐ Not changed<br>☐ Did not use tool for these foods

Butter; margarine
- ☐ *Did not consume*

- ☐ Increased
- ☐ Decreased
- ☐ Not changed
- ☐ Did not use tool for this food

Cooking oil, salad dressings
- ☐ *Did not consume*

- ☐ Increased
- ☐ Decreased
- ☐ Not changed
- ☐ Did not use tool for these foods

Alcoholic drinks
- ☐ *Did not consume*

- ☐ Increased
- ☐ Decreased
- ☐ Not changed
- ☐ Did not use tool for these drinks

Q11Finally, you may use the space on the next page for anything else you wish to tell us about your experience with this particular set of portion size tools.

This is the end of questionnaire.

THANK YOU VERY MUCH for your contribution.

Please return to your researcher at your next visit.

If you have any questions regarding this questionnaire or would like further clarification on how to fill it in please contact Amanda Aitken: a.aitken@chester.ac.uk or Professor Basma Ellahi – Faculty of Health and Social Care, University of Chester. Email address: b.ellahi@chester.ac.uk Telephone: 01244 512 177

S2a: Healthy Steps Instructions

Use of portion size tools for weight management

Directions on how to use the Healthy Steps serving set

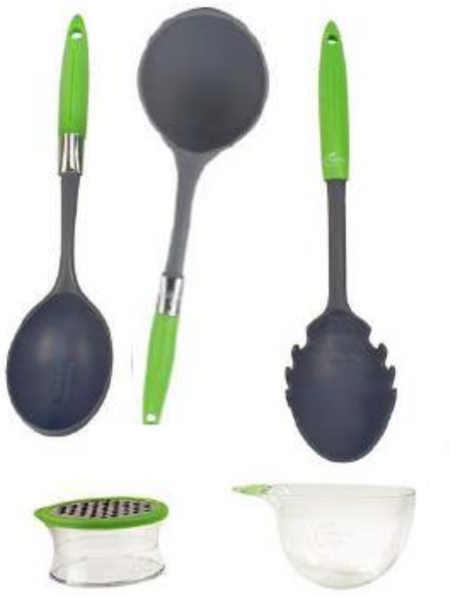

What are these tools?

This set of tools is manufactured by the Healthy Steps company and contains 5 components:

- **One solid spoon for starches** such as potatoes or rice (shown on the top left).
- **One** ladle for soup, gravy & sauce (shown in the top middle).
- **One pasta server** for cooked pasta & sauces (shown top left).
- **One cheese grater** measures one or two portions of cheese (shown bottom left)**.**
- **One cereal scoop measures one serving of cereal** (shown bottom right).

Each serving utensil measures a different amount of food (see below).

How to use Choose the appropriate tool for the food group to serve. For example:

1. **For starches** such as rice and potatoes, fill the solid spoon to serve one portion of potatoes or root vegetables (105 g), or rice/equivalents (80 g). Do not heap.
2. **For soup, sauce or gravy,** a ladle for cream-based sauce or gravy fill to the bottom gravy line, for a non-cream sauce fill to the middle sauce line for soup fill to the top mark (1 cup/1 serving).
3. **For cooked pasta** two spoons is a cooked pasta serving (200g/1 cup) and 2 scoops to the half way line for the pasta sauce.
4. **Cheese grater,** grate cheese to the middle line for one portion (1/4 cup =1 oz 31.5 and to the top for two (1/2 cup = 2oz = 63g).
5. **Cereal scoop** fill to the top for one portion of cereal (1 cup = 25g).

Heat resistance and cleaning information

These tools are nylon heat resistant to 400°F (204°C), and dishwasher safe.

Where can I get more help?

If you would like more information or assistance on how to use these tools as part of this study, please contact your **researcher Professor Basma Ellahi** – Faculty of health and Social Care, University of Chester, Parkgate Road, CH1 4BJ Email address:b.ellahi@chester.ac.uk Telephone: 01244 512 177.

Many thanks for your collaboration in this study

S2b: Precise Portions Instructions

Use of portion size tools for weight management

Directions on how to use the Precise Portions crockery set

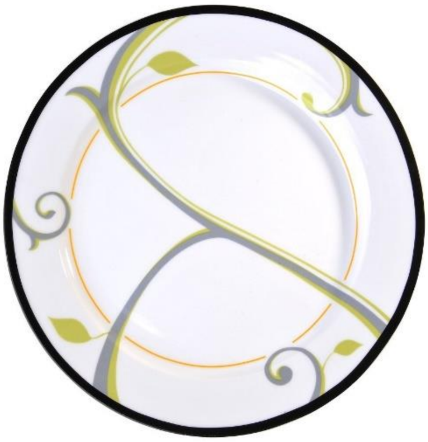

What are these tools?

This set of tools is manufactured by the Blue Horizon Development company and contains 2 components:

- **One 9-inch FOCUS plate** with sectors and discrete dietary guidelines printed on the plate

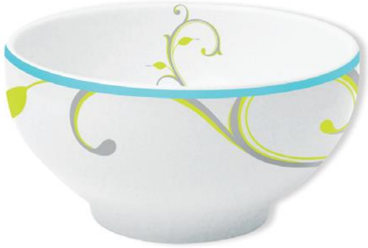

- **One 18 oz cereal/soup bowl** decorated with ½ cup, 1 cup and 1½ cups measurements integrated into a leaf motif design.

6. Serve non-starchy vegetables including cooked vegetables, raw vegetables and salad; meat, fish, egg-based dishes and meat-substitutes; and starches/grains on the corresponding sector of the Focus dish (it is advised not to heap the plate).
7. Use the bowl for breakfast cereals and soups. It can also be used for salads, fruit and other foods.

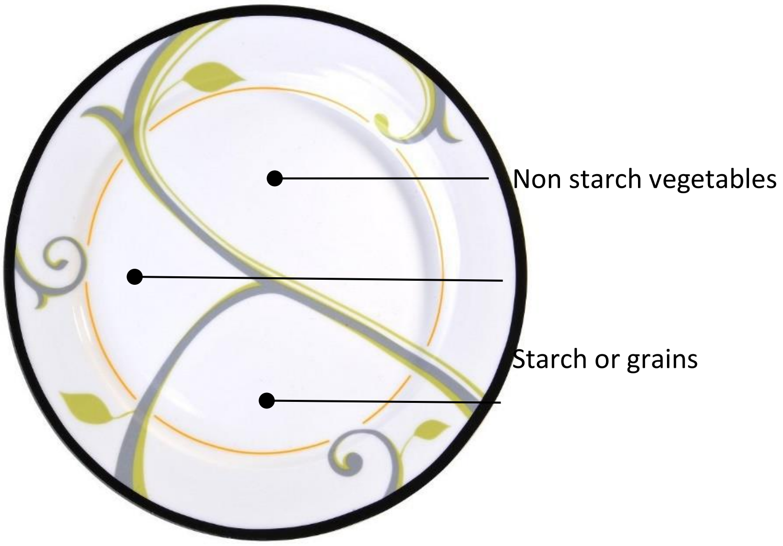

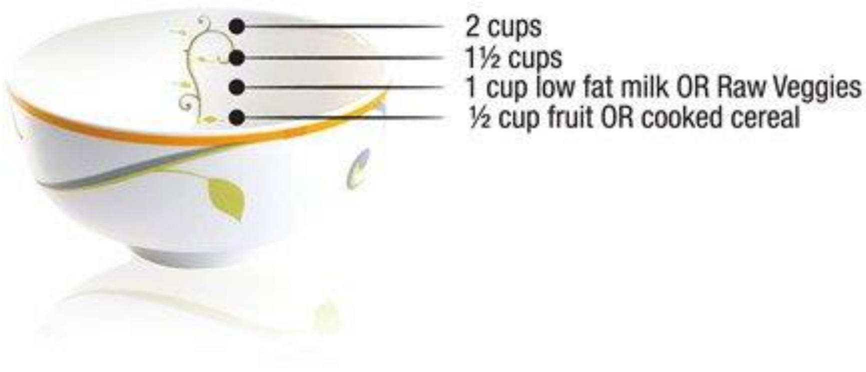

Heat resistance and cleaning information

These tools are made of chip-resistant porcelain and are lead and cadmium free. They are 100% dishwasher and microwave safe.

Where can I get more help?

If you would like more information or assistance on how to use these tools as part of this study, please contact your **researcher Dr. Basma Ellahi** – Faculty of health and Social Care, University of Chester, Parkgate Road, CH1 4BJ Email address: b.ellahi@chester.ac.uk Telephone: 01244 512 177.

Many thanks for your collaboration in this study.

S3: Analysis of Questionnaires 1,2 and 3

Scoring Scheme:

Acceptance was the mean score of questions 4a, 4b, 4c and reverse of 4d (liking, fitting the kitchen, fitting with home life and not feeling embarrassed using it), ease of use was calculated 4e, 4f, & 4g (easy use, resistant to wear and tear and having clear instructions, ease of use compared to other tool) and perceived efficacy was the average score of questions 4h, 4i, 4j & 4k. A score of 0.2,5 was considered a 'none - too low' result, a score between 2.6 – 3-4 as 'neutral' and between '3-5 to 'considered as moderate to high.
